# Analysis of the expression of Kv10.1 potassium channel in patients with brain metastases and glioblastoma multiforme: impact on survival

**DOI:** 10.1186/s12885-015-1848-y

**Published:** 2015-11-03

**Authors:** Ramón Martínez, Walter Stühmer, Sabine Martin, Julian Schell, Andrea Reichmann, Veit Rohde, Luis Pardo

**Affiliations:** 1 Department of Neurosurgery, University of Goettingen, Robert-Koch-Str. 40, Goettingen, 37075 Germany; 2 Department of Molecular Biology of Neuronal Signals, Max-Planck Institute for Experimental Medicine, Hermann-Rein-Str. 3, Goettingen, 37075 Germany; 3 Department of Neurosurgery and Neurotraumatology, Bergmannsheil Hospital, University of Bochum, Bochum, Germany

**Keywords:** Kv10.1, Potassium channel, Ion-channel, Brain metastases, Glioblastoma multiforme, Protein expression, Survival time, Tricyclic antidepressants, Tailored therapy

## Abstract

**Background:**

Kv10.1, a voltage-gated potassium channel only detected in the healthy brain, was found to be aberrantly expressed in extracerebral cancers. Investigations of Kv10.1 in brain metastasis and glioblastoma multiforme (GBM) are lacking.

**Methods:**

We analyzed the expression of Kv10.1 by immunohistochemistry in these brain tumors (75 metastasis from different primary tumors, 71 GBM patients) and the influence of a therapy with tricyclic antidepressants (which are Kv10.1 blockers) on survival. We also investigated Kv10.1 expression in the corresponding primary carcinomas of metastases patients.

**Results:**

We observed positive Kv10.1 expression in 85.3 % of the brain metastases and in 77.5 % of GBMs. Patients with brain metastases, showing low Kv10.1 expression, had a significantly longer overall survival compared to those patients with high Kv10.1 expression. Metastases patients displaying low Kv10.1 expression and also receiving tricyclic antidepressants showed a significantly longer median overall survival as compared to untreated patients.

**Conclusions:**

Our data show that Kv10.1 is not only highly expressed in malignant tumors outside CNS, but also in the most frequent cerebral cancer entities, metastasis and GBM, which remain incurable in spite of aggressive multimodal therapies. Our results extend the correlation between dismal prognosis and Kv10.1 expression to patients with brain metastases or GBMs and, moreover, they strongly suggest a role of tricyclic antidepressants for personalized therapy of brain malignancies.

## Background

Kv10.1 (Ether-à-go-go-1, KCNH1, Eag1) is a voltage-gated potassium channel, the expression of which is limited to selected brain areas such as hypothalamus, hippocampus, cerebral cortex, cerebellum and olfactory nerve [1]. It plays key roles in different physiological functions such as activation of excitable cells, hormone secretion regulation, cell to cell signal transduction, homeostasis of both blood pressure and osmoregulation of intracellular milieu [2]. Strikingly, Kv10.1 was also found to be a key player in regulation of cell division and proliferation [3] and overexpression has been detected at a very high rate (>75 %) in breast, renal and cervical carcinoma cell lines [4] as well as in different human malignancies, for instance colorectal [5] and cervical cancer [6], soft tissue sarcomas [7], acute myeloid leukemia [8], esophageal and gastric cancer [9, 10], head and neck carcinomas [11], ovarian [12], breast, lung and prostate cancer [13]. Aberrant expression of Kv10.1 has also been observed in regional lymph node metastases of gastric cancer and esophageal squamous cell carcinoma [10, 14]. Underscoring the oncological relevance of Kv10.1, previous analyses have recognized a correlation between the expression of Kv10.1 and patient prognosis. High Kv10.1 expression was associated with shorter overall survival of patients with esophageal and ovarian carcinomas [10, 12] as well as acute myeloid leukemia [8].

Although many efforts were made to unravel the role of Kv10.1 in cancer over years, the precise mechanisms remain only partially understood [15]. Previous investigations showed the relevance of Kv10.1 in cell cycle regulation [3] and proliferation control of tumor cells [4]. Laboratory data further indicated that aberrant expression of Kv10.1 is not an early event in pathogenesis, since aberrant Kv10.1 expression can be observed in experimental tumor models in which cancer had been triggered by further well established pathways [16]. A possible mechanism may be that Kv10.1 favors tumor progression through stimulating neo-angiogenesis via up-regulation of HIF-1 and VEGF in a tumor environment characterized by extreme hypoxia [17]. Loss of contact inhibition, accelerated proliferation [4] and increased migration [18] can also contribute to tumor progression, and therefore also non-solid tumors can benefit from Kv10.1 expression [8].

Since activity experiments of the Kv10.1 channel indicate cell membrane localization [6] the possibility to selectively block the channel was investigated. Blockade of Kv10.1 expression by specific monoclonal antibody [19] siRNA [20] or shRNA [21] led to reduced tumor cell proliferation and reduced tumor progression both in vitro [22] and in vivo [17, 19]. Furthermore, drug induced blockade of Kv10.1 expression, with the tricyclic antidepressant (TA) imipramine [22] and with astemizole in breast cancer cells [23], in both cases with IC_50_ in the low micromolar range, resulted in anti-tumorigenic effects. Furthermore, astemizole was found to increase calcitriol-induced antiproliferative activity in breast cancer by targeting Kv10.1, inhibiting CYP24A1 and up-regulating VDR [24]. Although ion channels are not the primary targets of imipramine or astemizole, both drugs block different channels with relatively high affinity by binding to intracellular regions; astemizole has been described to block several K^+^ channels related to Kv10.1, and imipramine blocks Na^+^, K^+^ and Ca^2+^ channels in different preparations [25].

Concerning GBM, only spare data with inconclusive results is available from the literature. Patt et al. [24] analyzed 5 GBMs and observed strong Kv10.1 expression in 3 out of 5 samples. Recently, Bai et al. [26] widely observed Kv10.1 overexpression in both GBM cell lines and clinical samples. To our knowledge, no investigation of Kv10.1 expression was previously performed in brain metastases. Since brain cancers represent the most frequent forms in adults and they are associated with a dismal overall survival, the necessity to identify selective therapies to improve the prognosis of the patients is mandatory.

In this study we have analyzed the expression of Kv10.1 in GBMs and in brain metastases from different carcinomas as well as the influence of Kv10.1 expression in survival. Moreover, we have analyzed the overall survival in GBM and brain metastasis patients who had undergone a post-operative therapy with tricyclic antidepressants, due to depression, and compared it with the OS of those patients who did not, and correlated these data with Kv10.1 expression.

## Methods

### Patients

Seventy-five consecutive patients with metastases to the brain from different carcinomas have been included. In 30 of them we have comparatively analyzed the Kv10.1 expression in the corresponding primary carcinoma as well. Furthermore, 71 patients with GBM were included for analysis of Kv10.1 expression. All patients were treated with tumor resection in the Department of Neurosurgery, University of Goettingen, Germany from 2004–2011, followed by adjuvant whole brain fractionated radiotherapy for brain metastasis or by focal radiotherapy for GBM (brain metastases: mean dose 35.8 Gray; GBM: mean dose 60 Gray) together with the alkylating drug temozolomide in the GBM cohort, according to neuro-oncological standard regimes [13].

Because of depression, 23/75 brain metastases patients and 26/71 GBM patients were additionally treated with antidepressants encompassing the tricyclic amitriptyline, the selective serotonin re-uptake inhibitors (SSRI) citalopram and sertraline as well as the tetracyclic mirtazapine (Table 1). Patients treated with additional long-term medication affecting the central nervous system (e.g. anticonvulsants) were not included in this study in order to avoid bias. Protocols and dosage of antidepressants had been chosen according to clinical standards. This study was performed with the approval of the local ethics medical committee, University of Goettingen (number 5/7/12). Written informed consent was obtained from the patient or patient caretaker.

**Table 1:** Scoring of Kv10.1 expression in brain metastasis patients regarding both localization of brain metastasis and type of primary carcinoma

Localization	n (%)	Kv10.1 score			
		0	1+	2+	3+
Cerebellar	24 (32 %)	2	11	10	1
Frontal	23 (30.5 %)	5	8	7	3
Parietal	12 (16 %)	3	1	6	2
Temporal	10 (13.5)	1	3	6	0
Occipital	6 (8 %)	0	2	1	3
Primary carcinoma					
Lung-carcinoma	37 (49 %)	7	11	13	6
Breast-carcinoma	14 (19 %)	2	8	4	0
Melanoma	6 (8 %)	1	1	3	1
Colorectal-carcinoma	4 (5 %)	0	1	3	0
Renal-cell-carcinoma	4 (5 %)	1	1	2	0
Ovarian-carcinoma	3 (5 %)	0	1	2	0
Prostate-carcinoma	2 (3 %)	0	1	1	0
Others	5 (7 %)	0	1	2	2

### Immunohistochemistry

For immunohistochemical analysis, formalin-fixed, paraffin-embedded tumor tissues were used. Immunohistochemical procedures were based upon formerly described protocols [27]. Briefly, tumor tissue was cut into 5 μm sections and mounted on silane-covered slides. After drying, sections were deparaffinized by rinsing in xylene two times for 10 minutes each, followed by hydration through an ethanol series (100-30 %, 5–2 min each). Antigen retrieval was performed by heating the slides for 30 min in 10 mM citrate buffer solution (pH: 6.0) at 90 °C in a water bath. After the slides cooled down to room temperature, non-specific binding sites were blocked using 10 % BSA in TBS for 1 h. For antigen detection, tissue sections were incubated with a recombinant single chain anti-Kv10.1 antibody fused to alkaline phosphatase (scFv62PhoA), in a dilution of 1:100 in TBS for 18 h at 24 °C. Subsequently, sections were washed 3x for 3 min with detection buffer solution containing 100 mM Tris-base, 100 mM NaCl and 5 mM MgCl_2_. Detection of alkaline phosphatase activity was performed by incubating the sections in BCIP/NBT (Roche Diagnostics, Rotkreuz, Switzerland) for 20 min. Finally, the sections were counterstained with Nuclear Fast Red (DAKO, Glostrup, Denmark), dehydrated and mounted with coverslips.

In order to double-check the former results, a second staining protocol was performed using the chromogen Neufuchsin with some modifications: antigen retrieval was performed by heating the slides for 30 min in a steamer at 60-70 °C in Tris-EDTA buffer (pH: 9.0). Non-specific binding sites were blocked with 0.2 % Casein for 20 min at room temperature. After the same antibody incubation as above, alkaline phosphatase activity was detected by incubating the sections in Neufuchsin solution (Sigma, Kawasaki, Japan), followed by counterstaining with Haematoxylin.

For immunohistochemical evaluation we used a Zeiss Axiovert 200 M inverted microscope (Carl Zeiss Microscopy GmbH, Goettingen, Germany), provided with a camera type Axiocam and Axiovision software. Non-linear adjustments were not used.

The same antibody has previously been used to characterize the distribution of Kv10.1 in human and murine brain [1]. As a positive control, we used cerebral tissue of adult C57/Bl6N mice to document the quality of antibody preparations. As negative control, sections were incubated with non-immune serum.

The stained tissue was analyzed semi-quantitatively using a score previously described [27] with some modifications as follows: Score 0, negative or less than 10 % of the tumor cells showed staining; Score 1+, faint staining in more than 10 % of the tumor cells; Score 2+, moderate staining in more than 10 % of the tumor cells; Score 3+, strong staining in more than 10 % of the tumor cells. Sections scored 0, 1+ were categorized as Kv10.1 low, sections scored 2+, 3+ as Kv10.1 high. The evaluation of the sections was performed by two experienced observers blinded to the patient diagnosis.

### Statistical analysis

The Kolmogorov-Smirnov-test was applied in order to assess the normal distribution of data. Analyses of differences in survival time of patients partitioned in groups according to Kv10.1 expression levels, antidepressant treatment and clinical parameters were performed with Student t-test or two-way ANOVA depending on the number of variables. Furthermore, survival studies were performed with the Kaplan-Meier analysis and the log-rank test.

The impact of Kv10.1 expression on survival time was evaluated using the Cox hazards regression analysis. For the Cox regression analysis, proportional hazards were considered. Proportionality was tested by the method of Grambsch and Therneau. We estimated the univariate effect on survival for each single Kv10.1 expression level and then we have included in the models major clinical predictors of outcome such as sex, gender, tumor localization, KPS (Karnofsky performance status) extent of surgical resection and RPA (recursive partitioning analysis, the last one for metastasis patients) and TA used, which allowed us control for the potential confounding effects, of these late variables. The final multivariate model included as covariates age, gender and tumor localization. Likelihood ratio tests were used to compare candidate models. A *p*-value <0.05 was considered statistically significant. Analyses were performed using Prism version 6 (GraphPad Software Inc., La Jolla, CA, USA) or SPSS Version 21 (SPSS Inc. der IBM Company, Chicago, USA) for the Kaplan-Meier analysis.

## Results

The male to female ratio was 1:0.9 in both GBMs and metastases collectives. The median age at diagnosis of patients with brain metastases was 60.7 years (SD: 12.7, range: 38–83 y.) and of patients with GBMs was 69.0 years (SD: 11.7, range: 30–84 y.).

### Analysis of survival in brain metastases and GBMs

Results of the univariate analysis in metastasis patients showed that better RPA class (I versus III and II versus III) was associated with improved survival (χ2 = 32.721, *p* = 0.01). In the group of GBMs, younger age of <45 y. (χ2 = 8.535, *p* = 0.01), KPS > 70 (χ2 = 19.763, *p* = 0.03) and extent of resection >98 % (χ2 = 21.765, *p* = 0.03) were also associated with longer survival of patients, as expected (log-rank test). In the multivariate Cox regression analysis of factors influencing survival in metastases, we have observed that low expression of Kv10.1 (*p* = 0.04; RR = 1.448; 95 % CI = 1.041-1.914) and better RPA class (*p* = 0.02; RR = 1.226; 95 % CI = 1.085-1.737) remained their prognostic significance. In glioblastoma patients, expression of Kv10.1 did not reach a prognostic significance as KPS, age and extent of tumor resection entered Cox’s regression model.

### Aberrant expression of Kv10.1 in brain metastases and GBMs

**Fig. 1:**
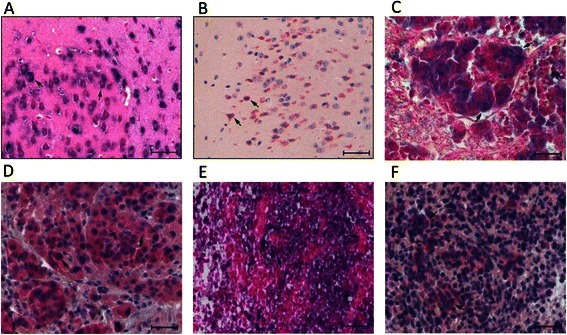
Examples of immunohistochemical analyses of Kv10.1 in brain metastasis and glioblastoma multiforme. **a**. NBT/BCIP staining of Kv10.1 (left) counterstaining nuclear fast red as positive control of Kv10.1 expression (adult C57BI6N mouse, cortical tissue) and **b**. Neufuchsin staining of Kv10.1 (right) counterstaining Haematoxylin (both magnification 400x, scale bar 50 μm). **c** NBT/BCIP staining of Kv10.1 (grade 2) in a brain metastasis of lung carcinoma (magnification 40x, scale bar 50 μm). **d** Neufuchsin staining of Kv10.1 (grade 2) in a brain metastasis of lung carcinoma, counterstaining Haematoxylin (both magnification 40x, scale bar 50 μm). **e**. NBT/BCIP staining of Kv10.1 in GBM (grade 3), counterstaining nuclear fast red, (magnification 40x, scale bar 50 μm). **f**. Neufuchsin staining of Kv10.1 (grade 3) in GBM, counterstaining Haematoxylin (magnification 40x, scale bar 50 μm)

A positive expression of Kv10.1 was observed in 64/75 (85.3 %) of brain metastases and in 55/71 (77.5 %) of GBMs. Expression scores and tumor localization in the brain as well as primary carcinoma of the brain metastases are shown in Table 1. Similarly, scores of Kv10.1 expression and tumor localization of the glioblastoma samples are provided in Table 2. Figure 1 shows microphotographs of Kv10.1 expression in selected brain metastasis and GBM, respectively.

**Table 2:** Kv10.1 expression scoring regarding brain localization of glioblastoma multiforme

Localization	n (%)	Kv10.1 score			
		0	1+	2+	3+
Temporal	26 (36.6 %)	5	7	13	1
Frontal	25 (35.2 %)	4	13	7	1
Parietal	18 (25.4 %)	6	7	5	0
Occipital	2 (2.8 %)	1	0	1	0

### Correlation of Kv10.1 expression and overall survival in brain metastases and GBMs

**Fig. 2:**
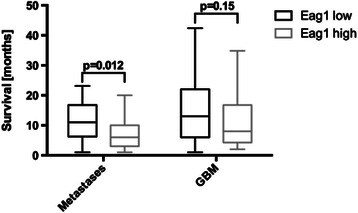
Association between survival and Kv10.1 expression in glioblastoma multiforme and brain metastasis patients. Analysis of overall survival in GBM and brain metastases patients depending on Kv10.1 expression revealing a significantly longer overall survival in those patients with brain metastases showing low expression of Kv10.1, as compared with brain metastases carrying a high Kv10.1 expression

Statistical analysis of the overall survival time showed that patients bearing brain metastases with a low expression of Kv10.1 had a significantly longer median survival time of 11 months (95 % CI: 7–13.7) compared to those patients displaying a high expression of Kv10.1, who had a median survival of only 6 months (95 % CI: 3 – 8.1, *p* = 0.012, Fig. 2). In the GBM collective, patients with a low expression of Kv10.1 had a median survival of 13 months (95 % CI: 9–17), whereas patients with a high expression of Kv10.1 showed a median survival of 8 months (95 % CI: 5–15.6, *p* = 0.15, Fig. 2).

### Correlation of Kv10.1 expression, treatment with antidepressants and overall survival

**Fig. 3:**
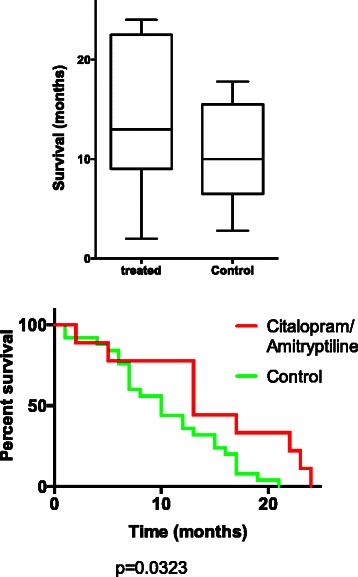
Relationship between Kv10.1 low-expression, therapy with antidepressants and overall survival in metastasis patients. Box-Whisker plot of median overall survival in brain metastases patients depending on Kv10.1 expression and antidepressants treatment therapy. A significantly longer median survival time in those patients with metastases with both low Kv10.1 expression and TA treatment is observed in the Kaplan-Meier analysis

**Fig. 4:**
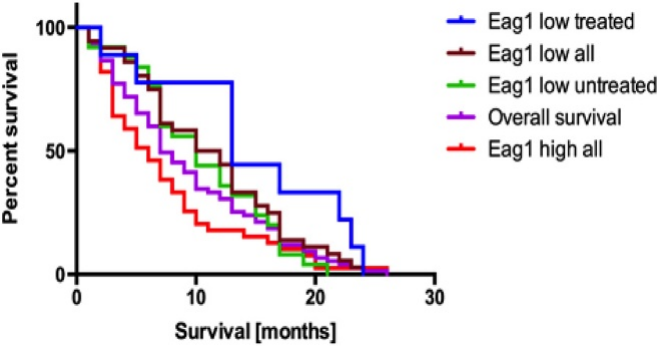
Analysis of survival in brain metastasis patients considering Kv10.1 expression and antidepressants therapy. Kaplan-Meier analysis of overall survival of patients with brain metastases depending on Kv10.1 expression and on antidepressant treatment. A significantly longer survival time in those brain metastasis patients with a low expression of Kv10.1 who had undergone treatment with antidepressants is observed

Brain metastases patients, displaying a low Kv10.1 expression and who had additionally undergone a treatment with antidepressants showed a significantly longer overall survival (median OS: 13 months, 95 % CI: 6.1-22.8) compared to untreated patients also displaying a low Kv10.1 expression (median OS: 10 months, 95 % CI: 7–14.7, *p* = 0.03, log-rank test, Fig. 3). This positive correlation could not be observed in brain metastasis patients with a high Kv10.1 expression also undergoing antidepressants treatment (median OS of treated patients: 6 months, 95 % CI: 3–9.9; median OS of untreated patients: 6 months, 95 % CI: 3–9, *p* = 0.1, log-rank test). Table 3 shows the univariate analysis of Kv10.1 expression, gender, tumor localization and survival in patients with brain metastases. Kaplan-Meier analysis of survival of brain metastasis patients showing differences in OS depending on expression of Kv10.1 and treatment with antidepressants is shown in Fig. 4. Furthermore, by multivariate Cox hazard analysis, a significant association was observed between low expression of Kv10.1 and longer survival time (*p* = 0.04, 95 % CI: 0.361-0.989). In contrast, the expression of Kv10.1 in GBM patients showed no significant influence on survival, independently of antidepressants therapy (*p* > 0.5, log rank test and Kaplan-Meier analysis, not shown) [24, 28].

**Table 3:** Univariate analysis of correlations between Kv10.1 expression, antidepressant therapy, gender and tumor localization in patients with brain metastases

Parameter	Number of cases	Survival (months)	*p*-value
	n (%)	median (95 % CI)	
Eag1 expression			
High	39 (52 %)	6 (3 – 8.1)	
Low	36 (48 %)	11 (7 – 13.7)	*0.012*
Antidepressants			
(amitriptyline/citalopram)			
Eag1 high			
Treated	8 (10.7 %)	6 (3 – 9.9)	
Untreated	27 (36 %)	6 (3 – 9)	0.1
Eag1 low			
Treated	10 (13.3 %)	13 (6.1 – 22.8)	
Untreated	25 (33.3 %)	10 (7 – 14.7)	*0.03*
Gender			
Male			
Eag1 high	26 (34.7 %)	6.2 (3.9 – 8.5)	
Eag1 low	13 (17.3)	10.6 (6.4 – 14.8)	*0.039*
Female			
Eag1 high	13 (17.3 %)	10.15 (6.1– 14.2)	
Eag1 low	23 (30.7 %)	11.65 (9 – 14.3)	0.5
Tumor localization of brain metastases			
Fronto-parietal			
Eag1 high	18 (24 %)	6.5 (3 – 10)	
Eag1 low	17 (22.7 %)	10 (7 – 16.9)	0.15
Temporo-occipital			
Eag1 high	10 (13.3 %)	6.5 (3 – 14.1)	
Eag1 low	6 (8 %)	8.5 (5.2 – 16.2)	0.6
Cerebellar			
Eag1 high	11 (14.7 %)	5 (2.8 – 8.2)	
Eag1 low	13 (17.3 %)	13 (5.6 – 16.4)	*0.03*

Significant *p*-values are italicized

### Comparative analysis of Kv10.1 expression in brain metastases and corresponding primary carcinomas

The expression of Kv10.1 was higher in brain metastases compared to the primary carcinomas in 60 % of the 30 analyzed matched pairs. It remained unchanged in 26.7 % and it was lower in 13.3 %. Analysis of groups of Kv10.1 expression showed significant differences regarding score 0 and 1 (see Methods for scoring; more frequent in primary tumors, *p* = 0.04 and 0.035, respectively) and score 2 (more frequent in brain metastases, *p* = 0.038). Survival of patients showing a low expression of Kv10.1 in both primary tumor and corresponding brain metastasis was significantly longer (*p* = 0.035).

## Discussion

Brain metastasis and GBM are the most frequent brain tumors in adults. In general, 6 % of patients with a newly diagnosed primary carcinoma will develop brain metastasis during cancer lifetime, based on USA data sets through Centers for Disease Control and Prevention and Surveillance, Epidemiology and End Results (SEER) Program [29]. This incidence is rapidly growing because of increasingly available diagnostic procedures allowing more accurate and earlier detection and because more efficient therapy modalities with better control of primary carcinoma and longer survival. The incidence of GBM based on data from the Central Brain Tumor Registry of the United States (CBTRUS, www.cbtrus.org) reaches 16 % of all primary CNS tumors and it is the most frequent astrocytic tumor (54 %) in adults. Both brain cancers are currently incurable. The median survival time of patients with brain metastasis based on the Recursive Partitioning analysis, RPA of the Radiation Therapy Oncology Group, RTOG [30] and on the Graded Prognostic Assessment, GPA [31] is 11 months after surgery and cranial radiotherapy. Furthermore, chemotherapy in brain metastasis plays only a secondary role, since large molecules developed for the treatment of primary carcinomas are not suitable to go through the blood brain barrier, making these malignancies inaccessible for currents drugs.

In the case of GBM, the scenario is also dismal with a median survival rate of 15 months in spite of multimodal treatment including gross-total resection, radiotherapy and chemotherapy, with the alkylating drug temozolomide, whereas the 2-year survival rate is below 14 % (CBTRUS). This survival rate is somehow higher in patients after gross-total resection of tumor, post-operative radiotherapy and concomitant chemotherapy with temozolomide, with GBM carrying promoter hypermethylation of the DNA repair gene *MGMT*, which occurs in 35-40 % of the cases [32, 33]. Taking this into consideration, every effort to delineate new therapeutic approaches is urgently needed.

Under physiological conditions, Kv10.1 expression is restricted to the central nervous system, and it is not normally expressed in differentiated peripheral tissues [1]. On the contrary, Kv10.1 is overexpressed in a variety of cell lines derived from human malignancies and in different cancers including head and neck, gastric, colon, hepatocellular pancreatic, renal or prostate carcinoma [4–6, 12, 14, 27] within which Kv10.1 enhances the proliferation of the cells and is required for the maintenance of growth. In these cases, Kv10.1 is not detected in the surrounding tissues.

One of the most striking characteristics of Kv10.1 is its relationship to cellular transformation. Kv10.1 channels are necessary for progression through the G1 phase and G0/G1 transition of the cell cycle [3]. Cells transfected with Kv10.1 lose contact inhibition, and induce aggressive tumors when implanted into immune-depressed mice [4]. Moreover, specific inhibition of Kv10.1 expression by the antisense technique, siRNA [20], or antibodies [19], leads to a reduction in tumor cell proliferation in vitro and in vivo. How overexpression of Kv10.1 occurs might be explained through deregulation of the pathway p53/miRNA34/E2F1. p53 negatively regulates Kv10.1 expression, thus inactivation of p53, as is the case in many cancers including secondary GBM, can cause oncogenic overexpression of Kv10.1 [34]. These findings support the molecular mechanisms associated with overexpression of Kv10.1 in tumor pathogenesis and add Kv10.1 to the p53/miRNA34/E2F1 regulator pathway with Kv10.1 mediating cell growth.

We have previously provided the link between the Kv10.1 channel and the mechanism to block this channel through drugs such as charged forms of antidepressants and astemizole which bind Kv10.1 to sites in the intracellular portion of the permeation pathway, only accessible when the channels are open (31). Tricyclic antidepressants (TA) such as imipramine, chlorimipramine, citalopram and amitriptyline have been previously reported to have anticancer properties [35–37]. Furthermore, cytotoxic effects have been demonstrated in various cancer cell lines including glioma cells [35–37] and colorectal cancer cells (35). Animal studies substantiate an anticancer action in various cancer experimental models, such as sarcoma and lymphocytic leukaemia [38, 39]. Jahchan [40] observed that TA induce apoptosis in small cell lung cancer (SCLC) cells in culture, and in mouse and human SCLC tumors transplanted into immuno-compromised mice. In these models, treatment with TA led to apoptotic cell death by activation of caspase-3, possibly through disruption of autocrine survival signals, even at doses used normally to treat depression. Moreover, the same apoptotic effect could be seen in high-grade neuroendocrine tumors, such as Merkel cell carcinoma, pheochromocytoma, and neuroblastoma.

Regarding gliomas and TA, a recent study suggested that the antidepressant desipramine could induce autophagy in C6 glioma cells through the PERK-ER (RNA–like endoplasmic reticulum kinase) stress pathway [41]. Imipramine has already been demonstrated to reduce cell proliferation, inhibit the PI3K/Akt/mTOR signaling pathway and to induce autophagic cell death in human glioma cells [42]. Furthermore, Levkovitz showed that selected antidepressants induce apoptosis in neuronal and glial cell lines by activation of p-c-Jun and subsequent increased mitochondrial released Cyt c [37].

To date, no data is available regarding expression of Kv10.1 in brain metastases. Furthermore, no molecular or clinical data is available concerning treatment with TA and survival in patients with brain metastases. In the present series we have expanded the significance of Kv10.1 in cancer to brain metastasis and GBM. Interestingly, in the case of brain metastasis, this phenomenon was independent of the histology of the primary carcinoma, suggesting that this event is related to the progression of disease, probably providing tumor cells a survival advantage under conditions frequently occurring in cancer, most probably hypoxia. A close consequence of hypoxia in cancer is an up-regulation of HIF-1, which is hallmark of cancer [43]. We had previously observed an increase in HIF-1 activity in Kv10.1-expressing cells, which represents a novel explanation for the oncogenic potential of Kv10.1 [17]. This hypothesis is further supported by the fact that the expression of Kv10.1 in brain metastases, compared to the expression in the corresponding primary carcinomas, was significantly higher in 60 % of the cases.

TA are currently used in clinical routine to treat a variety of diseases, such as major depression, neuropathic pain and fibromyalgia. TA were also shown to inhibit acid sphyngomyelinase (ASM), an enzyme catalyzing the hydrolysis of sphingomyelin to ceramide. Both, ASM and ceramide play an important role in different pathologies including diabetes, cystic fibrosis, major depression, Alzheimer’s disease and also in cancer. Blocking the synthesis of ceramide by inhibiting ASM introduced new therapy options for the treatment of the above mentioned diseases. In 2013, Peterson et al. reported that inhibition of acid sphingomyelinase selectively destabilizes cancer cell lysosomes, triggers cancer-specific lysosomal cell death, and reduces tumor growth *in vivo* [44]. Thus, cancer cells might fail to maintain sphingomyelin hydrolysis during exposure to ASM-inhibitors, such as tricyclic antidepressants, resulting in lysosomal destabilization due to sphingomyelin accumulation.

Sinergistic strategies of acid sphingomyelinase inhibition together with conventional chemotherapeutics and/or irradiation have been tested with promising results, also on glioma cells [45]. Nevertheless, a recent analysis showed that death of glioma cells after standard radio- and chemotherapy was not influenced by modulation of acid sphyngomyelinase and/ or glucosylceramide synthase pathway [46]. The last authors also observed a lack of association between modulation of the ceramide pathway and survival time of a large cohort of 564 studied patients with gliomas grade II, III and IV. This recent study has put into perspective the actual, probably less important significance, of the ceramide pathway in gliomas. Concerning brain metastases, no studies are available from the literature analyzing the influence of acid sphyngomyelinase on tumor progression or patient survival.

The translational impact of our results is highlighted by the observation that a significantly longer patient survival is associated with a lower Kv10.1 expression in the group with brain metastases, which confirms similar observations in non-CNS tumors, such as acute myeloid leukemia [8]. Contrastingly, we could not observe such a significant association in GBM. This observation would indicate a secondary role of Kv10.1 in GBM progression [24], although it does not preclude the potential relevance of the channel in other aspects of GBM, such as resistance to interferon [28]. One of the mechanisms via which overexpression of Kv10.1 contributes to tumor progression is an up-regulation of HIF1- and VEGF- mediated angiogenesis pathways. Taking into consideration that neo-angiogenesis is known to be up-regulated in GBM, one may argue that such up-regulation of angiogenesis associated factors is also achieved through Kv10.1 independent transduction signals.

Brain metastases patients showing a low Kv10.1 expression and treated with TA showed in our investigation a significantly longer overall survival compared to patients without TA therapy. In contrast, treatment with the antidepressant mirtazapine, a HERG (human Ether-à-go-go-Related Gene) channel blocker did not show this effect. These results strongly suggest that blocking Kv10.1 with TA in patients with low Kv10.1 expression might be relevant for tailored therapy of brain metastases. The fact that Kv10.1 blockade with TA are not significantly effective in patients with high Kv10.1 expression could indicate that the partial inhibition of Kv10.1 is not enough to alter the behavior of those highly malignant cases. Alternatively, it may be understood by taking into consideration previous results of our group [17]. Although no mutations in Kv10.1 have been reported in cancer, high expression of Kv10.1 may be linked with point mutations leading to conformational changes that could affect sensitivity to TA while maintaining intact its oncogenic potential, which is only partly dependent on ion permeation [17]. Nevertheless, this hypothesis needs further investigation.

## Conclusions

In summary, we have demonstrated for the first time that Eag1 is overexpressed in brain metastases of different primary carcinomas and that high Kv10.1 expression is associated with significantly poorer survival of patients. Moreover, inhibition of Kv10.1 with TA was associated with a significant longer survival time in brain metastasis patients and strongly suggests that Kv10.1 has a role in cell proliferation in these brain tumors as observed in non-CNS malignancies. These results underscore Kv10.1 as a potential tool in the tailored management of brain metastases and probably of glioblastoma multiforme as well.

## Abbreviations

GBM: Glioblastoma multiforme
OS: Overall survival
TA: Tricyclic antidepressant
siRNA: small interference ribonucleic acid
shRNA: small hairpin ribonucleic acid
CYP24A1: 1,25-dihydroxyvitamin D_3_ 24-hydroxylase
VDR: Vitamin D receptor
BSA: Bovine serum albumin
TBS: Tris buffered saline
mM: millimolar
BCIP/NBT: 5-bromo-4-chloro-3-indolyl phosphate/Nitroblue tetrazolium
Tris-EDTA: Tris-ethylene diamine tetraacetic acid
SD: Standard deviation
CI: Confidence interval
CNA: Central nervous system
MGMT: O-6-methylguanine-DNA-methyltransferase
HIF-1: Hypoxia inducible factor-1
VEGF: Vascular endothelial growth factor
RPA: Recursive partitioning analysis
KPS: Karnofsky performance score
